# Utilizing Polarization Diversity in GBSAR Data-Based Object Classification

**DOI:** 10.3390/s24072305

**Published:** 2024-04-05

**Authors:** Filip Turčinović, Marin Kačan, Dario Bojanjac, Marko Bosiljevac, Zvonimir Šipuš

**Affiliations:** Faculty of Electrical Engineering and Computing, University of Zagreb, 10 000 Zagreb, Croatia; filip.turcinovic@fer.hr (F.T.); marin.kacan@fer.hr (M.K.); dario.bojanjac@fer.hr (D.B.); marko.bosiljevac@fer.hr (M.B.)

**Keywords:** ground-based SAR, polarization, object classification, radar data, ResNet18

## Abstract

In recent years, the development of intelligent sensor systems has experienced remarkable growth, particularly in the domain of microwave and millimeter wave sensing, thanks to the increased availability of affordable hardware components. With the development of smart Ground-Based Synthetic Aperture Radar (GBSAR) system called GBSAR-Pi, we previously explored object classification applications based on raw radar data. Building upon this foundation, in this study, we analyze the potential of utilizing polarization information to improve the performance of deep learning models based on raw GBSAR data. The data are obtained with a GBSAR operating at 24 GHz with both vertical (VV) and horizontal (HH) polarization, resulting in two matrices (VV and HH) per observed scene. We present several approaches demonstrating the integration of such data into classification models based on a modified ResNet18 architecture. We also introduce a novel Siamese architecture tailored to accommodate the dual input radar data. The results indicate that a simple concatenation method is the most promising approach and underscore the importance of considering antenna polarization and merging strategies in deep learning applications based on radar data.

## 1. Introduction

Advancements in affordable microwave technology in recent years have allowed for the research and development of various small radar systems, spawning many new possibilities. In addition, further rapid development of these systems has been achieved through integration with deep learning algorithms and tools to bring new smart sensing applications to life. Synthetic aperture radar (SAR) systems are an important representative in this sector as active microwave imaging sensor types used to generate radar images which can be efficiently processed by the tools of artificial intelligence. Combining multiple SAR images (i.e., multispectral or multi-polarization) in such applications offers additional input to the algorithms and can enhance the accuracy of the application task, as it has been demonstrated in several recent works [1,2].

In particular, the developments of Ground-Based Synthetic Aperture Radars (GBSARs) have significantly been impacted by these advancements [3,4]. GBSAR is a terrestrial remote sensing system that can provide high-resolution images of small areas. It utilizes the SAR concept of sensor antenna motion to virtually extend the sensor antenna aperture by acquiring radar signals from multiple positions along its path. At each of these positions, the sensor extracts information about the distance, which is later used to reconstruct a two-dimensional radar image of the observed area. Unlike satellite or airborne SAR systems, it is typically used for applications which require stable and accurate measurements such as surface deformation, dam and landslide monitoring, stability assessment. and other cases [3,4,5,6,7]. Additionally, GBSAR systems offer space for significant parameter optimization, depending on whether the goal is system efficiency, accuracy, or something else. One of the parameters that can be easily used and changed if necessary is antenna polarization. Research focused on utilizing the information obtained from different polarizations is well known and has been conducted in various SAR applications. For instance, in [8,9], authors extract polarization properties from various terrain targets, while in [10], single-polarization and dual-polarization radar data are compared in the hail detection. Recently, investigations of various polarization in GBSAR applications have also shown potential, for example, when dealing with circular polarizations [11,12] or using polarimetric information for target classification [13].

In recent years, deep learning models have efficiently utilized the availability of such multilayered data from sensors for various signal processing tasks. Different deep learning models with SAR and GBSAR databases are being used [14] to advance the understanding and potential of these systems, and in final applications help monitor and understand complex physical processes [15]. However, the drawback in these applications often lies in small datasets corresponding to a particular application. This means that the performance of deep learning models is highly dependent on the input data and the radar parameters used to acquire these data. On the other hand, the advantage of GBSAR systems is that we can have access to the raw data (signal data which are normally used for generating reconstructed radar images). Access to raw data by itself can be very beneficial in deep learning classification applications since these data are not modified by reconstruction algorithms [16]. Additional layers of such data, for example, data obtained using different polarization, then allow for new options in utilizing those data in subsequent processing and classification. Usually, SAR has two reconstructed images available for different polarizations later combined in processing, which can be computationally intensive. However, if we use raw signals recorded using different polarizations, we can combine these signals before deep learning processing and potentially make the final classification less computationally intensive. This aligns with our goal, as we aim to develop energy-efficient radar systems based on low-power microprocessors.

In this paper, we investigate the potential of utilizing polarization information to enhance the performance of deep learning models for object classification. Based on our earlier research with the GBSAR-Pi system developed by our group, we have explored object classification using radar data. Now, our study aims to analyze how different methods can utilize orthogonal polarization data to improve object classification through deep learning techniques. Specifically, we want to compare the classification outcomes of models trained on input data generated by applying various merging methods to the raw GBSAR data obtained using both horizontal and vertical polarizations. We conduct a set of recordings of observed scenes containing several objects of different material and shape, recorded using two orthogonal polarizations, to evaluate the effectiveness of incorporating such information in a classification model. The deep learning model for the classification task is based on ResNet18 [17] architecture which is modified to operate with raw GBSAR data. The classification results of various polarization combinations are used to provide insights into their impact on radar data and, consequently, object classification. Additionally, we introduce a novel deep learning architecture based on the modified ResNet18, which utilizes two inputs separately: raw data obtained with horizontal and vertical polarization. The results show that with optimal use of polarization data, both classification accuracy and energy efficiency of the system can be improved. We note that besides straightforward comparison of merging methods of different polarization data for improving classification in signal-based applications, similar ideas of combining two or more kinds of data in the form of matrices can be utilized to enhance various deep learning tasks.

The rest of this paper is structured as follows. In Section 2, we present a GBSAR system for object classification. Section 3 describes the data acquisition process, including the experiment setup, original datasets generated using the GBSAR-Pi system developed by our group, and additional datasets generated by aforementioned various merging methods. The results of the classifications based on the listed datasets and their interpretation are given in Section 4. Finally, we provide a discussion of the implications of our results in Section 5.

## 2. Object Classification Using GBSAR Data

GBSAR, analogous to SAR, virtually extends sensor antennas by leveraging sensor movement along a ground-based track while emitting and receiving EM waves, enabling distance extraction and radar image reconstruction. The Frequency-Modulated Continuous Wave (FMCW) radar principle is commonly used as a sensor in such systems due to its simple implementation [5,18]. A FMCW radar emits a continuous signal with a changing frequency, sweeping through a defined band *B*. Since this frequency sweep is also present in the echo signal delayed by travel time, mixing it with the emitted signal enables the extraction of delay using low-frequency differences, which is then used to calculate the distance to the target [19]. Sensor movement can be in a continuous or a stop-and-go mode, with the latter allowing precise selection of step and aperture length, impacting azimuth resolution. Range resolution, on the other hand, is determined by the sensor’s bandwidth. Hence, smaller step length and wider bandwidth provide more information about the observed scene and better overall resolution of the reconstructed radar image. Additionally, the polarization of the EM waves emitted by the sensor depends on the orientation and type of the sensor antennas. This parameter is commonly utilized in SAR systems for discriminating various targets [20].

Typically, using the distance information from multiple steps allows for us to reconstruct a 2D radar image, which we can interpret more easily, and use such obtained image in further processing. However, in applications where we have access to raw radar data (signals), this is not always necessary since modern AI tools also allow for us to exploit the raw data directly. This was demonstrated in [16], where comparison of object classification based on raw data and reconstructed images was given. It was shown that using raw data as input for deep learning had equal or even better classification results. The added benefit was that there was no need to initially perform radar image reconstruction, which can be time consuming for larger datasets.

**Figure 1:**
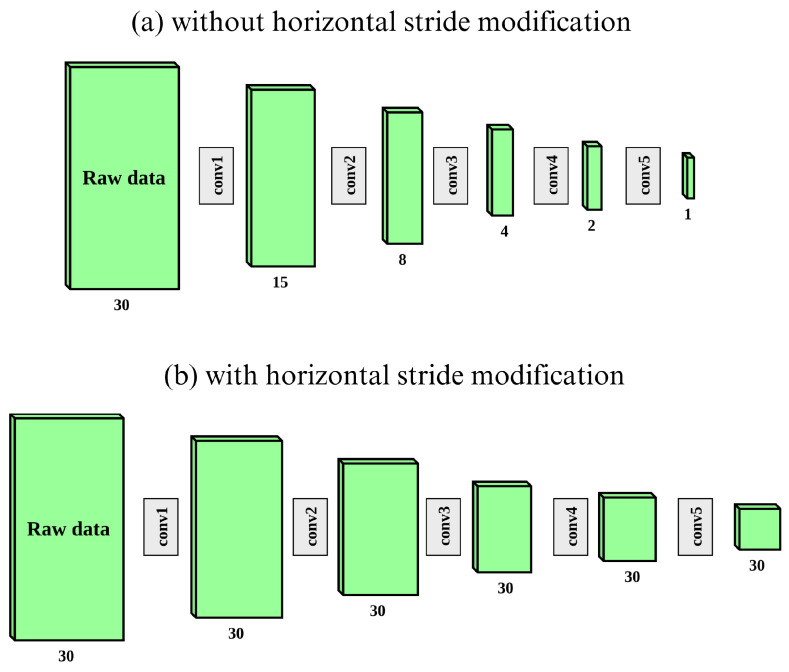
ResNet18 modification for raw GBSAR data [16]. In (**a**), the matrix dimensions reduce after each conv group, while in (**b**) (with modification), the horizontal dimension remains the same throughout the process.

The used model is based on ResNet18 architecture with an ajdustment to raw GBSAR data. In the case of a smaller number of steps which corresponds to the dimensions of one axis in the resulting matrix, the architecture is modified by removing downsampling steps in that axis. Specifically, in our case, each column of the resulting matrix represents the signal obtained after mixing from one sensor (the FMCW module) position along the track. Therefore, the dimension of the vertical axis depends on the number of frequency points of that signal which is, in our case, 1024. The number of such signals in the resulting matrix (or the number of GBSAR steps) represents the horizontal spatial dimension and is, in our case, 20. In Ref. [16], it was shown that on occasions in which one spatial dimension is much larger than the other, it is beneficial to remove the downsampling steps of the smaller axis. This is achieved by setting the horizontal stride to one, which prevents the model from halving the horizontal dimension in each of the five convolution groups from regular ResNet18 architecture. The proposed modification is displayed in Figure 1. All classification models in this paper are based on ResNet18 with mentioned modification.

Finally, from the application perspective, after the classification process, the system has to make a decision whether classification results are satisfactory or not, and for this we need to evaluate a probability distribution over all possible classes. This approach was presented in [21] where the feedback algorithm for an energy-efficient GBSAR system is given. The proposed algorithm consists of two object classifications: initial and final, where the initial one is based on GBSAR recordings conducted using a smaller number of steps. The classification process results in the mentioned probability distribution, and the class with the highest probability is taken as the predicted one. If that probability is higher than the set limit, the observed example is considered classified. Otherwise, if the probability is lower than the limit, the recording of that object is conducted from additional positions in the second GBSAR measurement, after which the final classification is run. This way, the system initially reduces energy consumption by classifying simpler examples using fewer steps. In deep learning applications, this probability distribution is often calculated using softmax function [22],

(1) $$f_{s}\left(z_{k}\right)=\frac{e^{z_{k}}}{\sum_{j=1}^{K}z_{j}}$$

where $z_{k}$ represents the logit output of the model for one class. In that approach, the question of whether softmax function can determine the model’s certainty arises. In order to answer it, we analyze the results of our classification models trained with GBSAR data recorded using horizontal and vertical polarization.

## 3. Data Acquisition

### 3.1. Experiment Setup

**Figure 2:**
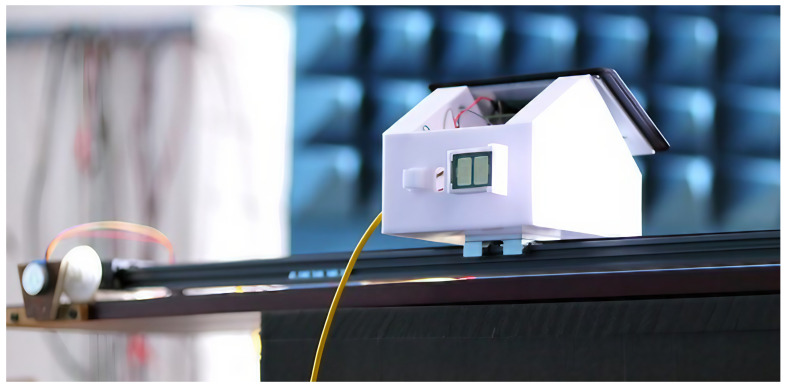
Developed GBSAR-Pi [16].

**Figure 3:**
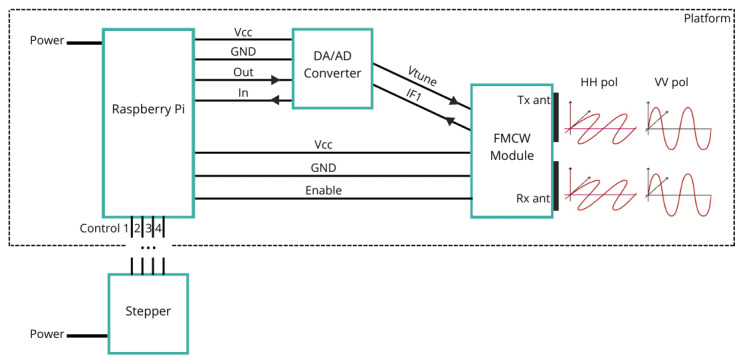
Scheme of GBSAR-Pi [24]. GBSAR-Pi is based on microcomputer Raspberry Pi and FMCW module. The module can emit horizontally or vertically polarized EM waves.

The measurements are conducted using the GBSAR-Pi system described in [16]. GBSAR-Pi is implemented around Raspberry Pi, operates in a stop-and-go mode, and utilizes the FMCW module Innosent IVS-362 [23] to capture the resulting signal from each position. It is implemented around microcomputer Raspberry Pi. The radar in the real-world setup is shown in Figure 2, while its schematic display is given in Figure 3. The platform housing the microcomputer, the AD/DA converter, and the FMCW module is moved along a 1 m long track by a 5 V stepper motor. With a central frequency of 24 GHz and a frequency bandwidth (*B*) of 1.3 GHz, the sensor’s antennas are integrated into the module, resulting in their polarization being determined by the module’s orientation. The polarization change in this study is manually executed by rotating the module before recordings, enabling the acquisition using single polarization (HH or VV); however, in practical applications, two modules with orthogonal polarizations are used in parallel.

The scenes are observed from 60 positions spaced 1 cm apart, meaning that the original matrix has dimensions of 1024 × 60. In each of the observed scenes, three objects are set at an approximately 30 cm distance from the radar. The matrices are later split into three segments, each of which covers one object where the dimensions of each segment are 1024 × 20. The reason behind the splitting is the potential utilization of the aforementioned feedback algorithm which enhances the energy efficiency of the system and primarily focuses on the impact of additional polarization data when using such low-resolution data. All scenes are first recorded using horizontal polarization and then using vertical polarization, resulting in a total of 50 pairs of measurements per polarization.

### 3.2. Original Datasets

Since in all 100 conducted recordings, the scenes contained three objects, the datasets consist of 300 data points. Hence, there are 150 data points recorded with horizontal polarization in the ‘HH’ (horizontal) dataset and 150 data points recorded with vertical polarization in the ‘VV’ (vertical) dataset. Data points are matrices of dimensions 1024 × 20.

The observed objects were approximately of same size but made of different materials (metal, glass, plastic) and had different shapes (cylinder and cuboid). Also, the width and height of these objects were comparable so that the response to neither polarization could be potentially dominant. The orientation of the cuboids was perpendicular to the direction of the emitted EM waves. Each of the six combinations of materials and shapes are represented by one object and each object stands for one class. The six classes and their marks are as follows:

- Po—plastic cylinder;
- Pc—plastic cuboid;
- Go—glass cylinder;
- Gc—glass cuboid;
- Mo—metal cylinder;
- Mc—metal cuboid.

**Figure 4:**
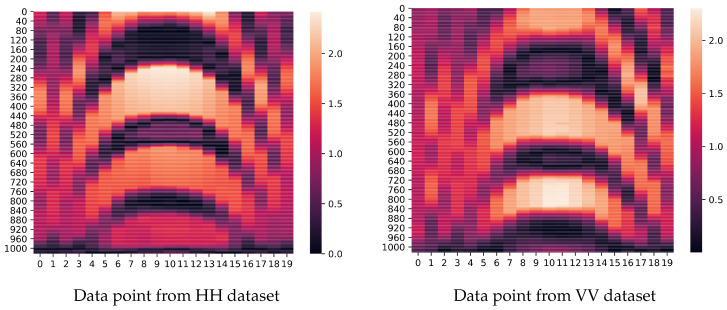
Examples of raw radar data recorded with horizontal (**left**) and vertical (**right**) polarization. Observed scene contained a metal cuboid (Mc).

It is expected to receive signals with the highest intensity from metal objects and signals with the lowest intensity from plastic ones. The shape of the objects should impact the adjacent signals around the center of the object. The heatmaps of one pair of the same object (metal cuboid) in HH and VV datasets are shown in Figure 4. The dataset including HH and VV data points is publicly available.

### 3.3. Additional Datasets

In this study, we analyze the results of the models trained on datasets obtained with radar recordings using different polarizations. In two consecutive recordings, all scenes were initially captured using horizontal polarization, followed by vertical polarization. Besides unprocessed ‘HH’ and ‘VV’ matrices which represent data obtained with horizontal and vertical polarization (respectively), we create additional datasets by combining the following pairs:

- SUB: Dataset examples generated by subtracting the matrix recorded with vertical polarization from the one recorded with horizontal polarization.
- AVG: Dataset examples generated by averaging matrices recorded with vertical and horizontal polarizations.
- MIX_ROWS: Dataset examples generated by alternately mixing rows of matrices recorded with horizontal and vertical polarizations.
- MIX_COL: Dataset examples generated by alternately mixing columns of matrices recorded with horizontal and vertical polarizations.
- JOIN: Dataset examples generated by appending the matrix recorded with vertical polarization to the end of the matrix recorded with horizontal polarization.

**Figure 5:**
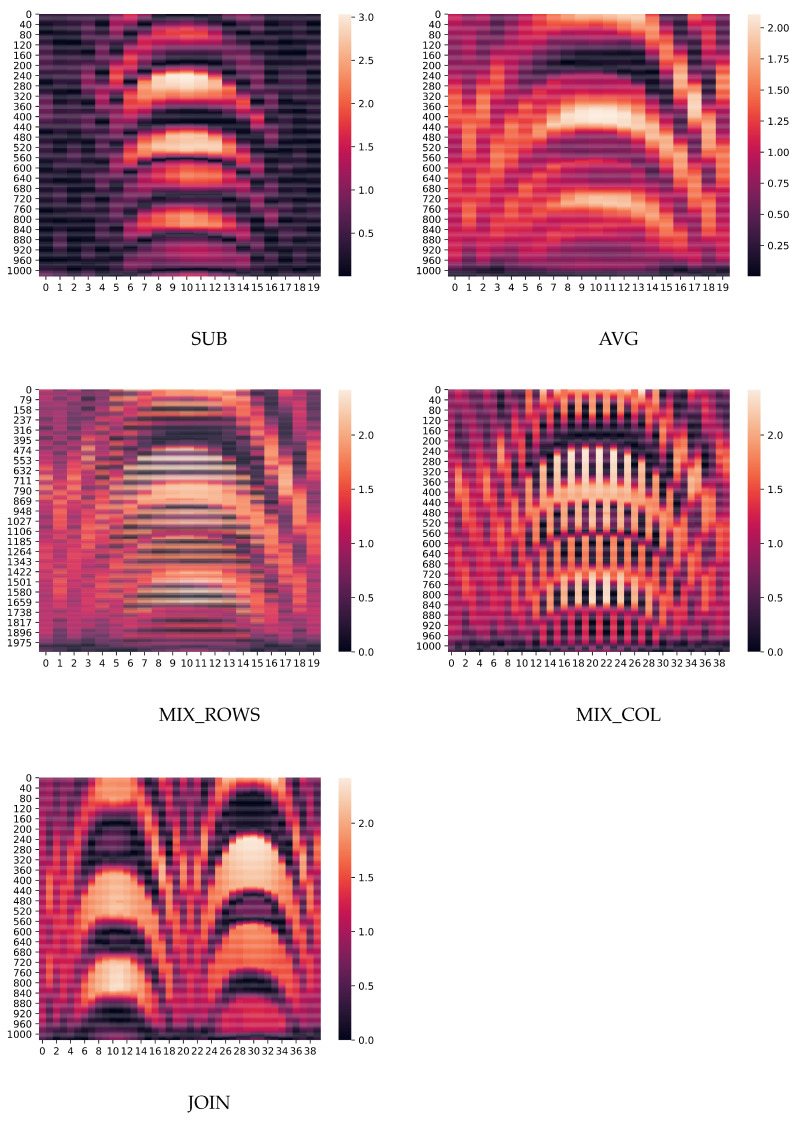
Examples of the datasets: SUB, AVG, MIX_ROWS, and MIX_COL i JOIN. The examples are the combinations of the matrices depicted in Figure 4, i.e., they represent a metal cuboid object.

The combinations contain the same data elements of the two matrices (horizontal and vertical) presented in different ways. In the first two combinations (SUB and AVG), the matrix elements are calculated by subtracting or averaging the elements from the two original matrices so the dimensions of the resulting matrix, for example, from these datasets, are the same as the originals. The elements of the three remaining datasets are the same as the elements from the original matrices but placed in different matrix positions. Therefore, their dimensions are doubled in either horizontal or vertical axes. The dimensions of all matrices from the listed datasets are provided in Table 1. Figure 5 displays the heatmaps of examples from each dataset. The idea of generating such datasets is to test which of them is best suited for the classification task, i.e., a model trained on which dataset will achieve the highest accuracy.

## 4. Results

A distinct model was trained using each dataset, culminating in the creation of a total of seven deep learning models that underwent testing over ten iterations. To ensure a fair comparison of the approaches, during each iteration, the models were trained, validated, and tested on the same examples. For instance, if a horizontal polarization model (HH) was tested on the example ‘Mc_3_hh_48’, representing a cuboid metal object set in the third position, captured with horizontal polarization in the measurement scene number 48, the corresponding test examples for vertical polarization model (VV) included ‘Mc_3_vv_48’, while test set of other models contained, for example, ‘Mc_3_48’, which is generated using the aforementioned combinations of ‘Mc_3_hh_48’ and ‘Mc_3_vv_48’. We note that models are named after the datasets they are trained with.

**Figure 6:**
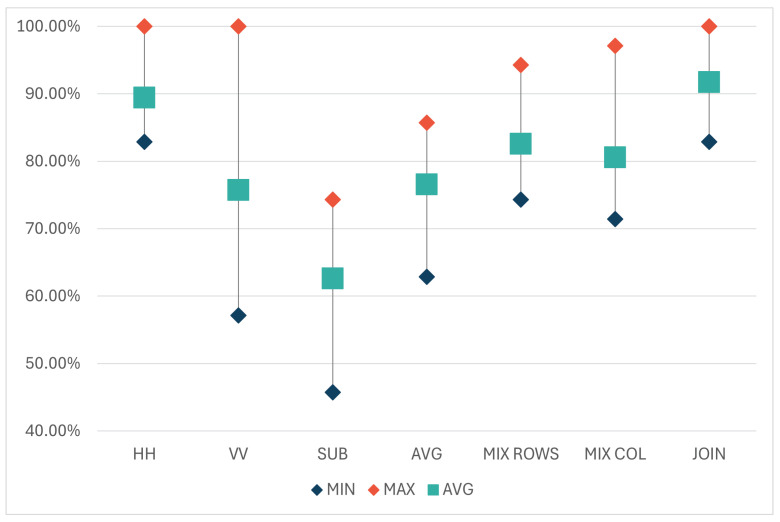
Minimum, maximum, and average classification accuracy of the HH, VV, SUB, AVG, MIX_ROWS, MIX_COL, and JOIN models.

Table 2 (and Figure 6) presents the minimum, maximum, and average accuracies over 10 iterations for each model. The results indicate that the models trained on the data obtained with horizontal polarization were, on average, more accurate than those trained on the data using vertical polarization (89.43% compared to 75.71%). Both models achieved 100% accuracy in the same iteration. However, in eight out of the nine remaining iterations, the model trained on the HH dataset outperformed the VV model. A possible reason behind this discrepancy can be the selection of test objects and their positions or orientations in the scene. Hardware limitations of the utilized FMCW module (Innosent-362 [23]) may also have contributed, as its radiation pattern, according to technical specifications, exhibits different behaviors in the horizontal (45°) and vertical (38°) axes. The main point to note is that HH and VV models yield different results, indicating they are trained on distinct information. Thus, integrating both polarizations could potentially enhance accuracy.

Regarding the combinations, the model trained on the SUB dataset, where matrices are subtracted from each other, yielded the weakest performance. This outcome was expected, as the subtraction process removes a part of the information. Similarly, the AVG dataset, where information is lost through matrix averaging, also achieved lower accuracy results. The second group of combinations (MIX_COL, MIX_ROWS, and JOIN) consists of information from both original matrices (horizontal and vertical polarizations), hence it was anticipated that they would perform better than the first two combinations. In all three datasets, the same elements from the HH and VV matrices were used but arranged differently. The results show that models trained on MIX_COL and MIX_ROWS datasets produced similar results (with averages of 80.58% and 82.57%, respectively), while the JOIN dataset, where the original matrices are placed side by side, yielded the highest results compared to all trained models, averaging 91.72%.

These results can be observed from a perspective beyond radar signal processing, considering the influence of the order of elements within the same matrix on the classification outcome of the model based on the same architecture. In the JOIN dataset, the order of elements remains the same as in the original matrices, preserving information about the positions and arrangement of individual signals alongside their values. This is in contrast to MIX_COL and MIX_ROWS datasets, where the original matrices are fragmented into columns or rows, respectively. The potential drawback of the JOIN dataset is that signals obtained at the same position with horizontal and vertical polarizations in the JOIN matrix are separated by the width of one matrix (the number of GBSAR steps). Specifically, in our case, the first column of the JOIN matrix is recorded from the same position as the 21st column but with different polarizations. On the other hand, in the MIX_COL dataset, two adjacent columns represent signals recorded from the same position, but the order within the two original matrices is disrupted, leading to a degradation in classification accuracy, as indicated by the results. In MIX_ROWS, both matrices are similarly fragmented by rows, again reducing classification accuracy. Hence, the JOIN model, which provides the best results, has the advantage of preserving the order of the elements within matrices. However, it faces the challenge of treating two concatenated matrices as one, where the distances between columns do not represent equal spatial distances. To address this drawback, several alternative approaches were analyzed in a second set of 10 iterations with Ensemble and Siamese models.

### 4.1. Ensemble and Siamese Models

**Figure 7:**
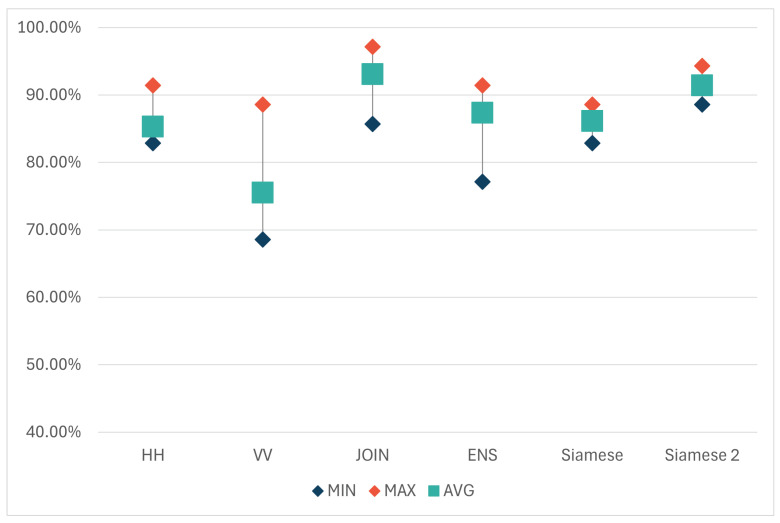
Minimum, maximum, and average accuracy of HH, VV, JOIN, Ensemble, Siamese and Siamese 2 models.

First, we combined the results of models with horizontal (HH) and vertical (VV) matrices into an Ensemble model. In the Ensemble model, the softmax results of HH and VV models are averaged to obtain the final softmax result (Equation (1)) for each class. The accuracy of the Ensemble model, denoted as ‘ENS’ hereafter, directly depends on the outcomes of HH and VV models. In cases where both models make errors on the same examples, those examples are likely to be misclassified in the ENS model. However, there have been cases in which the opposite occurred. In one such case, the horizontal model misclassified an object of class ‘Go’ as ‘Mo’, while the vertically polarized model classified it as ‘Gc’. Thus, the HH model erred in material but correctly recognized the shape, while the VV model correctly identified the material but made a mistake in classifying the shape. On the other hand, as both models predicted ‘Go’ as the second most likely class with high probabilities, the ENS model correctly classified the example. The results over the second set of 10 iterations, depicted in Table 3 and in the form of a graph in Figure 7, once again showed a higher accuracy of the HH model compared to the VV one. On average, the horizontal model successfully classified 85.31% of the test set examples, while the vertical model achieved this for 75.51% of examples. Interestingly, the ENS model, on average, outperformed the results of both individual models. Out of 10 iterations, the ENS model was more accurate in 6, tied with the horizontal model in 3, and was only worse once. In that case, the vertical model decreased the accuracy of the ENS.

Since we established in the first analysis that in our case, scenario HH model outperforms the VV model, an alternative version of the Ensemble model was tested. In this version, due to the superiority of the HH model, the influence of this model on the averaging of results was increased by multiplying its class probabilities by a factor greater than one when calculating the probabilities for the new Ensemble model. However, this approach improved the Ensemble model’s result in only one iteration, while in the others, it achieved the same accuracy.

**Figure 8:**
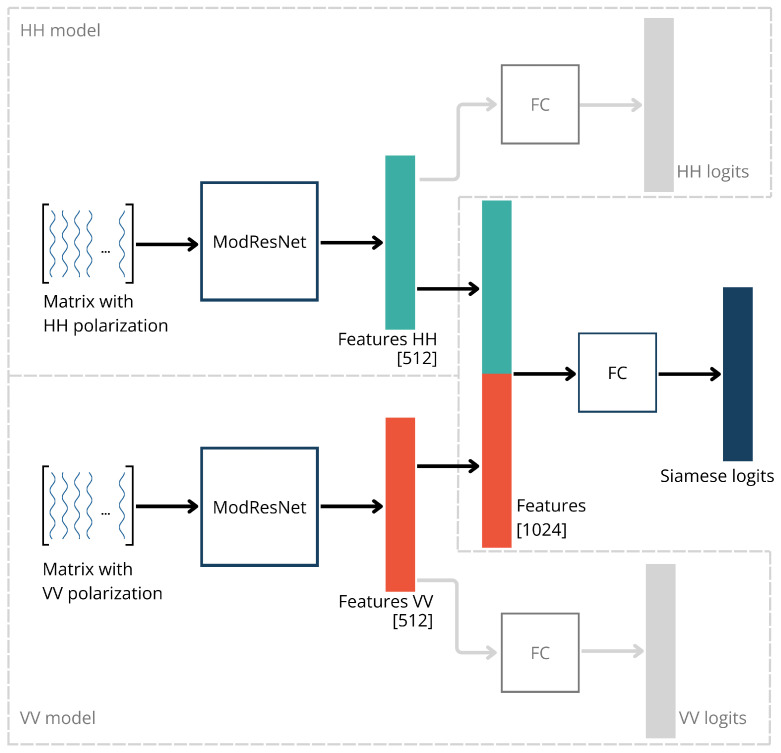
Architecture of the Siamese model with two separate branches for HH and VV matrices. Each branch functions as a distinct HH or VV model. The branches connect their feature vectors to form a Siamese feature vector, which is then fed into a fully connected layer.

On the other hand, the results once again imply that the combination of the HH and VV matrices can be utilized to improve classification results. Therefore, we tested another approach in which two classification branches (one for each polarization) were trained in parallel. Each branch had an architecture similar to the ModResNet model, receiving a matrix of one polarization as input. Before the fully connected (FC) layer of the ModResNet models, the branches connected their feature vectors (each of size 512) in one feature vector (of size 1024) of the entire model, which was then fed into a fully connected (FC) layer. Subsequently, similar to the original model, probabilities for each class were calculated, and predictions were made. This model is hereafter referred to as the ’Siamese’ model, and its architecture is illustrated in Figure 8.

That way, the Siamese model can train two parts of the network (one for each polarization) and eventually connect the feature vectors to determine the class by learning from both matrices separately. Although this approach, on average, yielded slightly better classification results than models trained on a single polarization, there was no significant improvement. Such an outcome was not expected since Siamese is a more complex network trained with more data compared to HH and VV models. However, it is worth noting that the dataset used in all these models is still relatively small, which, especially in the case of complex networks, can negatively impact classification results due to insufficient training samples to set the weights properly.

To address this, we tested the same approach with initialized weights for both sub-models (branches). The weights for the part of the network trained on matrices for horizontal polarization were set using the weights of the pre-trained horizontal model, and the part trained on matrices for vertical polarization used the weights of the pre-trained vertical model. The weights were reset in each iteration to correspond to the examples used to train the horizontal and vertical models. This model is hereafter referred to as ‘Siamese 2’. Such approach increased the average accuracy when compared to regular Siamese from 86.12% to 91.43%, but it required training of HH and VV models in order to initialize weights. Interestingly, even that model, on average, did not outperform the JOIN model. The reason for this could be the aforementioned limited dataset, which is better suited to simpler models, and the fact that the shared vector before the fully connected layer is created by combining two vectors of the same size which may be considered to have an equal influence of both polarizations on the final accuracy, which, as shown earlier, is not the case, as horizontal polarization consistently provided more accurate results in all iterations. In the second set of 10 iterations, the Siamese 2 model was better than the JOIN model in three, equally accurate in one, and worse in six cases. Figure 7 displays the results of all models in the second set of 10 iterations, where, in addition to HH, VV, and JOIN, the tested models include the Ensemble model ENS, as well as Siamese and Siamese 2.

An additional argument for employing the JOIN approach is the network’s simplicity. Single ResNet18 models, including variants like HH, VV, and JOIN, are computationally simpler compared to Siamese models due to their streamlined processing of a single input. In single ResNet18 models, all computations are performed within a single branch, which simplifies both the model’s architecture and the training process. The Siamese models require the processing of two inputs through two separate branches, which doubles the number of network parameters, resulting in higher computational load. When comparing it to other deep learning architectures used in SAR data-based models, it is worth mentioning that LSTM (Long Short-Term Memory) and GCNs (Graph Convolutional Networks) introduce additional layers of complexity. LSTMs are often used in SAR automatic target recognition [25,26]. However, their recurrent nature and ability to capture temporal dependencies contribute to this added complexity. GCNs, on the other hand, are utilized for graph-structured data like SAR images [27,28], leveraging graph convolution operations to capture spatial relationships among data points which can also be computationally expensive.

### 4.2. Softmax Function Evaluation

Since the Softmax function in the implementation of the feedback algorithm described in [21] is used to determine the confidence of the model, we evaluated it over all 20 iterations performed in this paper. The output probabilities of all 4200 examples were grouped into percentage bins: 0–50%, 50–60%, 60–70%, 70–80%, 80–90%, and 90–100%. The number of correct predictions was then compared with the total number of predictions in each bin. The results, presented in Table 4 show that the accuracy of each bin falls within that percentage range, thereby confirming the previously stated thesis regarding the use of the softmax function as a measure of model confidence.

### 4.3. Polarization Impact on the Classification of the Specific Object

As we have shown before, models based on horizontal polarization matrices achieved better classification results compared to those based on vertical polarization. Specifically, in the first 10 iterations, the difference in the average accuracy of these two models was around 14 percentage points (75% versus 89%), while in the second set of iterations, a slightly lower difference of 10 percentage points was observed (75% versus 85%). In only one iteration out of 20, the VV model achieved a higher accuracy than the HH model.

To examine the impact of polarization on specific test objects in recordings, we analyzed all 20 iterations of both horizontal and vertical models, along with the JOIN model, given its superior performance among the assessed approaches. For each test object, we compared the number of test examples that were correctly classified and, when they were not, we determined the object they were most frequently mistaken for. It is noteworthy that the observed scenes featured objects composed of three different materials (metal, glass, and plastic) and two distinct shapes (cylinder and cuboid).

In the case of horizontal polarization, all objects except the plastic cylinder (64.17%) were classified with an accuracy above 85%. The plastic cylinder was misclassified as a plastic cuboid in 33% of cases, with negligible misclassifications with other classes. The plastic cuboid was correctly classified in 89.17% of cases, with no standout misclassification compared to others. On the other hand, although 86.67% and 89.17% of glass and metal cuboids, respectively, were correctly classified, when they were not, the glass cuboid was most often mistaken for the metal cuboid, and vice versa. As for the remaining two objects (glass and metal cylinder), they were correctly classified in more than 97% of cases when recorded with horizontal polarization. The confusion matrix of the HH model is shown in Table 5.

In the case of vertical polarization, only the metal cuboid was correctly classified in more than 85% of cases, while the lowest accuracy, similar to the HH model, was achieved for the plastic cylinder (48.33%). In 45% of cases, class was misclassified for the plastic cuboid, and the plastic cuboid was most often misclassified for the glass cylinder. On the other hand, when metal and glass objects were misclassified, it was mostly due to the material rather than the shape. The correct and predicted classes over 20 iterations for vertical polarization are given in Table 6.

**Figure 9:**
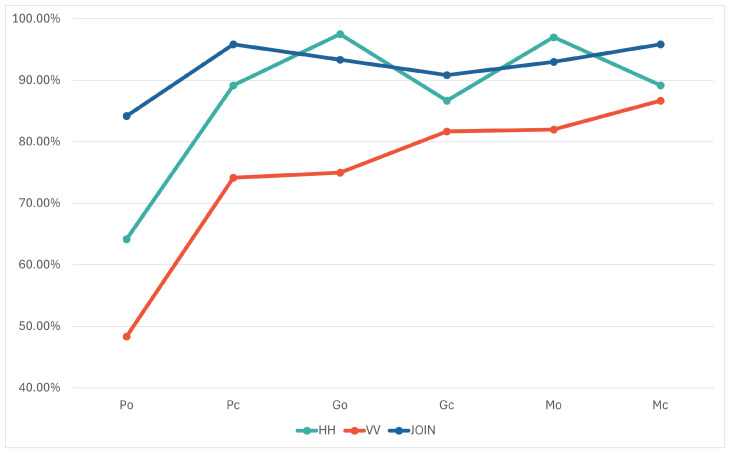
Comparison of the average accuracy per class of HH, VV, and JOIN models.

In the analysis of the JOIN approach, all objects were classified with an accuracy over 90%, except for the plastic cylinder (84.17%), which was most often mistaken for the plastic cuboid. The results of this model for all classes over 20 iterations is given in Table 7. A comparison of all three approaches by classes is shown in Figure 9.

## 5. Conclusions

In this paper, we explored the potential of utilizing orthogonal polarization information in the context of deep learning object classification based on ground-based synthetic aperture radar (GBSAR) data. Our study focused on comparing the classification results of models trained on (original) datasets in which data points (in form of a matrix) are obtained using different polarizations in the GBSAR system. Furthermore, we employed various merging methods to generate additional datasets using combinations of original ones including subtraction, averaging, mixing rows and columns, and concatenation.

The results revealed that, in our case scenario, the models trained on data obtained with horizontal polarization (HH) consistently outperformed those trained on data obtained with vertical polarization (VV). Specifically, the HH model, on average, reached 13 percentage points greater classification accuracy than the VV model in the first set of test iterations (89% compared to 76%), and 9 percentage points greater in the second one (85% to 76%). Further analysis of the merging methods demonstrated that the concatenation of matrices, as implemented in the JOIN dataset, yielded the highest classification accuracy in both sets, averaging 92% and 93%. The results indicate that the JOIN dataset preserved the order of elements within matrices, contributing to improved classification results. Such outcome can also be observed from a perspective beyond classification in radar data, since the idea of concatenating two matrices with different information as in the JOIN approach may be utilized in other deep learning applications.

We also explored Ensemble and Siamese architectures to incorporate the information obtained using both polarizations in the same model. The Ensemble model, directly combining the results of the horizontal (HH) and vertical (VV) models, showed improved performance, surpassing the individual models in most iterations. The Siamese model, which trained two branches (one for each polarization) separately before merging them into one feature vector, which is forwarded to the fully connected layer, exhibited a similar outcome to those of individual and Ensemble models, indicating potential benefits from such an approach. To enhance the complexity, in the next approach, we introduced the Siamese 2 model in which the weights were initialized using pre-trained HH and VV models. While Siamese 2 achieved higher accuracy than the previous models (91% in average), it did not consistently outperform the simpler JOIN model, emphasizing the importance of dataset size.

To summarize, our findings suggest that the careful consideration of the antenna polarization and merging strategies of data obtained with differently polarized EM waves emitted by sensors in the data acquisition process can significantly impact the accuracy and efficiency of deep learning models in radar applications. Specifically, the JOIN merging method, with its simplicity and effectiveness, emerged as a promising approach, demonstrating the potential for improved classification accuracy and energy efficiency of GBSAR systems based on low-power microprocessors. The Siamese architecture also exhibited notable potential; nevertheless, to fully realize its capabilities, a more extensive dataset is essential.

## Figures and Tables

**Table 1:** The dimensions of the examples of the original and generated datasets. ‘HH’ represents the dataset with matrices obtained using horizontal polarizations, ‘VV’ represents the one using vertical polarizations.

HH	VV	SUB	AVG	MIX ROWS	MIX COL	JOIN
1024 × 20	1024 × 20	1024 × 20	1024 × 20	2048 × 20	1024 × 40	1024 × 40

**Table 2:** Minimum, maximum, and average accuracies over 10 iterations for each model trained on the following datasets: HH, VV, SUB, AVG, MIX_ROWS, MIX_COL, and JOIN. The highest average accuracy is marked bold.

	HH	VV	SUB	AVG	MIX ROWS	MIX COL	JOIN
Min.	82.86	57.14	45.71	62.86	74.29	71.43	82.86
Max.	100	100	74.29	85.71	94.29	97.14	100
Average	89.43	75.71	62.6	76.57	82.57	80.58	**91.72**

**Table 3:** Minimum, maximum, and average classification accuracy of the HH, VV, JOIN, Ensemble, Siamese, and Siamese 2 models. The highest average accuracy is marked bold.

	HH	VV	JOIN	ENS	Siamese	Siamese 2
MIN	82.86	68.57	85.71	77.14	82.86	88.57
MAX	91.43	88.57	97.14	91.43	88.57	94.29
AVG	85.31	75.51	**93.06**	87.34	86.12	91.43

**Table 4:** Correctly classified examples for each percentage bin.

Bin	Correctly Classified	Total in Bin	Percentage
0–50	137	293	46.76%
50–60	202	361	55.96%
60–70	245	371	66.04%
70–80	371	467	79.44%
80–90	593	678	87.46%
90–100	1906	2027	94.03%

**Table 5:** Confusion matrix of the HH model. Classes are marked as Po—plastic cylinder, Pc—plastic cuboid, Go—glass cylinder, Gc—glass cuboid, Mo—metal cylinder, Mc—metal cuboid.

		Predicted Class							
		**Po**	**Pc**	**Go**	**Gc**	**Mo**	**Mc**	**Total**	**Accuracy**
**True class**	**Po**	77	40	0	1	2	0	120	64.17%
	**Pc**	1	107	1	4	3	4	120	89.17%
	**Go**	0	0	117	2	1	0	120	97.50%
	**Gc**	0	2	0	104	0	14	120	86.67%
	**Mo**	0	0	3	0	97	0	100	97.00%
	**Mc**	0	0	0	13	0	107	120	89.17%
	**Total**	1	42	4	20	6	18	700	

**Table 6:** Confusion matrix of the VV model. Classes are marked as Po—plastic cylinder, Pc—plastic cuboid, Go—glass cylinder, Gc—glass cuboid, Mo—metal cylinder, Mc—metal cuboid.

		Predicted Class							
		**Po**	**Pc**	**Go**	**Gc**	**Mo**	**Mc**	**Total**	**Accuracy**
**True class**	**Po**	58	54	0	8	0	0	120	48.33%
	**Pc**	7	89	13	2	9	0	120	74.17%
	**Go**	8	5	90	7	10	0	120	75.00%
	**Gc**	2	6	2	98	1	0	120	81.67%
	**Mo**	0	4	12	2	82	11	100	82.00%
	**Mc**	0	0	0	14	2	104	120	86.67%
	**Total**	17	69	27	33	22	18	700	

**Table 7:** Confusion matrix of the JOIN model. Classes are marked as Po—plastic cylinder, Pc—plastic cuboid, Go—glass cylinder, Gc—glass cuboid, Mo—metal cylinder, Mc—metal cuboid.

		Predicted Class							
		**Po**	**Pc**	**Go**	**Gc**	**Mo**	**Mc**	**Total**	**Accuracy**
**True class**	**Po**	101	18	0	1	0	0	120	84.17%
	**Pc**	0	115	0	0	2	3	120	95.83%
	**Go**	3	1	112	0	4	0	120	93.33%
	**Gc**	0	4	2	109	0	5	120	90.83%
	**Mo**	2	5	0	0	93	0	100	93.00%
	**Mc**	0	0	0	4	1	115	120	95.83%
	**Total**	5	28	2	5	7	8	700	

## Data Availability

The data is available at “Ground Based SAR Data Obtained With Different Polarizations”, Mendeley Data, V1, doi: 10.17632/nbc9xpwv96.1, online: https://data.mendeley.com/datasets/nbc9xpwv96 (accessed on 8 February 2024).

## Abbreviations

The following abbreviations are used in this manuscript:

SAR	Synthetic Aperture Radar
GBSAR	Ground Based SAR
FMCW	Frequency-Modulated Continuous Wave
B	Frequency bandwidth
EM	Electromagnetic
CNN	Convolutional Neural Network
ModResNet	Modified ResNet18 model
FC	Fully connected layer
